# Supplementary material for: Food hardness preference reveals multisensory contributions of fly larval gustatory organs in behaviour and physiology
Source: PLoS Biol. 2025 Jan 30;23(1):e3002730. doi: 10.1371/journal.pbio.3002730 (PMC11781724; doi:10.1371/journal.pbio.3002730)
Supplement: S1 Table — (DOCX) [file pbio.3002730.s005.docx]

**Supplementary table 1: Fly stocks used**

| **Genotype** | **Source** | **Stock Reference** |
| --- | --- | --- |
| GMR57BO4-Gal4 | BDSC | 46355 |
| Gr94a-Gal4 | BDSC | 57686 |
| Gr97a-Gal4 | BDSC | 57687 |
| UAS-myr::GFP | BDSC | 32198 |
| UAS-GCaMP6m | BDSC | 42748 |
| Gr21a-Gal4 | BDSC | 23890 |
| Gr63a-Gal4 | BDSC | 9942 |
| pain-Gal4 | BDSC | 27894 |
| nan-Gal4 | BDSC | 24903 |
| nompC-Gal4 | BDSC | 36361 |
| UAS-rpr | BDSC | 5824 |
| nSyb-Gal4 | BDSC | 68222 |
| UAS-Gr21a-RNAi | VDRC | 104122 |
| UAS-Gr63a-RNAi | VDRC | 108203 |
| UAS-pain-RNAi | VDRC | 39477 |
| UAS-nan-RNAi | VDRC | 100090 |
| UAS-nompC-RNAi | VDRC | 330013 |
| UAS-tmc-RNAi | VDRC | 110911 |
| UAS-H2B::RFP | ^1^ | N/A |
| UAS-H2B::YFP | ^2^ | N/A |
| Peb:T2A::Gal4^DBD^ ; ; pb::T2A::P65^AD^ | This study | N/A |

1. Langevin, J. *et al.* Lethal Giant Larvae Controls the Localization of Notch-Signaling Regulators Numb, Neuralized, and Sanpodo in Drosophila Sensory-Organ Precursor Cells. *Current Biology* **15**, 955–962 (2005).

2. Bellaïche, Y., Gho, M., Kaltschmidt, J. A., Brand, A. H. & Schweisguth, F. Frizzled regulates localization of cell-fate determinants and mitotic spindle rotation during asymmetric cell division. *Nat Cell Biol* **3**, 50–57 (2001).
