# Supplementary material for: Food hardness preference reveals multisensory contributions of fly larval gustatory organs in behaviour and physiology
Source: PLoS Biol. 2025 Jan 30;23(1):e3002730. doi: 10.1371/journal.pbio.3002730 (PMC11781724; doi:10.1371/journal.pbio.3002730)
Supplement: S2 Table — (DOCX) [file pbio.3002730.s006.docx]

**Supplementary table 2: Primers used for generation of the split-*GAL4* lines (restriction sites underlined, CRISPR sites blue)**

| Name | Sequence |
| --- | --- |
| T2A Xho fw | AGCTCGAGGGCAGAGGCAGCCTG |
| p65AD H3 re | GGAAGCTTACTTGCCGCCGCC |
| zipper Start BamH fw | AAGGATCCCTGGAGATCCGCGCCGC |
| Gal4 DBD H3 re | ATAAGCTTTTACGATACCGTCAGTTGCCG |
| Peb CDS Kpn fw | ACGGTACCGCATCTGGTGGACCCCATG |
| Peb end Xho re | GCCTCGAGGGCGGAGGAGAGCAGC |
| Peb 3’UTR fw | CAATCGAGCGACACCAAGAGCC |
| Peb 3’UTR Xba re | AGTCTAGAGTTGCTGCTGCTGCATCCAC |
| Peb CRISPR1 fw | TATATAGGAAAGATATCCGGGTGAACTTCGACGAGGCGGCCAAACTTCTGTTTTAGAGCTAGAAATAGCAAG |
| Peb CRISPR2 re | ATTTTAACTTGCTATTTCTAGCTCTAAAACTACGTATACCAAAGCTTAACGACGTTAAATTGAAAATAGGTC |
| Pb CRISPR1 fw | TATATAGGAAAGATATCCGGGTGAACTTCGCAACTCAGTTAGTTCATGTGTTTTAGAGCTAGAAATAGCAAG |
| Pb CRISPR2 re | ATTTTAACTTGCTATTTCTAGCTCTAAAAC﻿GAGGCCAGTGTAAATATTTCGACGTTAAATTGAAAATAGGTC |
| Pb ex Kpn fw | CAGGTACCTCACCAGCCAGCGGC |
| Pb end Xho re | ATCTCGAGACTGAGTTGGTAGTATTCCGGCGC |
| Pb 3’UTR H3 fw | TAAAGCTTAGCCTAATTCGCCGCGTGGG |
| Pb down Xba re | AACTCCTCTAGAGCCGTGGCCTAGAC |
