# Supplementary material for: Food hardness preference reveals multisensory contributions of fly larval gustatory organs in behaviour and physiology
Source: PLoS Biol. 2025 Jan 30;23(1):e3002730. doi: 10.1371/journal.pbio.3002730 (PMC11781724; doi:10.1371/journal.pbio.3002730)
Supplement: S3 Table — (DOCX) [file pbio.3002730.s007.docx]

**Supplementary table 3: One sample t and Wilcoxon statistical analysis of GO ablation behavioural experiments compared with a 0 mean.**

| **Test** | **Condition** | **N** | **Mean** | **OSS p-value** | **Significance** |
| --- | --- | --- | --- | --- | --- |
| **Sucrose v Plain** | **GO > rpr** | **13** | **0.04103** | **0.5461** | **ns** |
|  | **GO x yw** | **13** | **0.3436** | **<0.0001** | ******** |
|  | **Rpr x yw** | **13** | **0.441** | **0.0013** | ****** |
| **Quinine v Plain** | **GO > rpr** | **13** | **0.04103** | **0.7342** | **ns** |
|  | **GO x yw** | **13** | **-0.4308** | **<0.0001** | ******** |
|  | **Rpr x yw** | **14** | **-0.3571** | **0.0006** | ******* |
| **EtAc v H2O** | **GO > rpr** | **13** | **0.559** | **<0.0001** | ******** |
|  | **GO x yw** | **13** | **0.4462** | **0.0021** | ****** |
|  | **Rpr x yw** | **13** | **0.7282** | **<0.0001** | ******** |
| **Light v Dark** | **GO > rpr** | **13** | **-0.5744** | **<0.0001** | ******** |
|  | **GO x yw** | **13** | **-0.4821** | **<0.0001** | ******** |
|  | **Rpr x yw** | **13** | **-0.4308** | **0.0005** | ******* |
| **1% vs 2.5%** | **GO > rpr** | **13** | **0.1026** | **0.2026** | **ns** |
|  | **GO x yw** | **13** | **0.359** | **<0.0001** | ******** |
|  | **Rpr x yw** | **13** | **0.3641** | **0.0002** | ******* |
| **1% vs 0.1%** | **GO > rpr** | **13** | **-0.1795** | **0.0073** | ****** |
|  | **GO x yw** | **13** | **0.4308** | **<0.0001** | ******** |
|  | **Rpr x yw** | **13** | **0.2923** | **0.0165** | ***** |
