# Supplementary material for: Food hardness preference reveals multisensory contributions of fly larval gustatory organs in behaviour and physiology
Source: PLoS Biol. 2025 Jan 30;23(1):e3002730. doi: 10.1371/journal.pbio.3002730 (PMC11781724; doi:10.1371/journal.pbio.3002730)
Supplement: Attachment — Submitted filename: Revisions_TouchMS_CF.docx [file pbio.3002730.s008.docx]

Reviewer #1: Komarov et al. investigate the role of gustatory chemosensory neurons and mechanosensation in feeding in larval Drosophila. First, they describe that larvae prefer intermediate substrate hardness - they avoid very soft and very hard agar. To understand which taste neurons play a role in this behavior, they constructed a split GAL4-line, which specifically labels many gustatory receptor neurons in the fly larva. Ablating all these cells leads to deficits in sugar and quinine sensing but does not affect odor attraction and phototaxis. Larvae with ablated GRs also cannot differentiate substrate hardness anymore. Knocking down mechanosensors in the larvae revealed that painless receptors are required for hard substrate avoidance and proper feeding. Painless positive neurons also responded to mechanical stress and one of them also to sucrose. The authors identified the C6 neuron as being responsive to sucrose and mechanical stimulation. This neuron is also receptive
to CO2 and expresses GR63a and GR21a. Ablating the CO2 GRs did not affect sucrose nor mechanical response, but the cells did not respond to CO2 anymore. Ablation of the C6 cell or knockdown of the painless receptor did not affect substrate preference, suggesting that other cells can compensate for the behavior.

The authors describe a new role for gustatory sensory cells, indicating that they are involved in substrate hardness sensing. They can further show that many GRs respond to mechanical stimulation and a broad ablation of GRs impairs softness sensing.
The current study provides further evidence that many chemosensory cells not only express a single receptor and might be broadly tuned to different cues. The authors should cite also the relevant literature for multisensory coding in a single sensory neuron in the adult fly:
- Vosshall lab, Herre et al., 2022 - mosquito sensory neurons express multiple sensors
- Potter lab, Task et al., 2022 - ORNs express a multitude of receptors, such as IRs and GRs

*This is indeed a good point. We have included these citations in the introduction to contextualise receptor coexpression as previously described (line 65).*

The authors show that the C6 neuron responds to CO2, sucrose and mechanosensory stimulation, does it also respond to other tastants? Also, is it known which sensors mediate the sucrose responsiveness?

*The C6 neuron was previously shown to be multimodal to tastants, responding to high salt, glycerol, sucrose, among others. This was characterised in van Giesen et al., 2016 Although the receptor mediating the sucrose response has not been identified.. We have added this information to the paragraph describing known properties of C6 (lines 404-407)).*

*The section reads as follows: “Furthermore, C6 has previously been shown to be multimodal, responding to high salt concentration, sucrose, glycerol, and HCl among substances tested using a calcium imaging approach.”*

Is the painless channel required for the neuronal response to mechanical stimulation in C6 - do they lose their response in functional imaging? This data would be relevant to show that painless is the softness receptor. However, other neurons might compensate for the behavioral stability even under C6 ablation (Figure 4D'). The authors do not describe this lack of phenotype in the results.

*Indeed, we find that painless knockdown does not significantly affect the mechanical response, suggesting that while the channel may be present in the neuron, it does not affect either physiology or behavioural responses to mechanical stimulation or substrate hardness preference. We have emphasised these results in the text (lines 385-391) and in the legend of figure 4. Additionally, we have added a section of discussion to speculate whether C6 is relevant for mechanosensitive behaviours, or whether other neurons are able to compensate in its absence (lines 416-420).*

Reviewer #2:
MAJOR:
The use of the term "texture" in this work seems incorrect. More precise terminology is needed. For example, the different agar here has different material properties which are well described by terms such as compressibility, shear modulus,  rigidity, elasticity or stiffness, but not texture as I typically understand it.  I most associate the term texture with the surface profile of a material, so  I would have expected the paper to explore the animal's ability to detect  "bumpiness" or "roughness" of food surfaces: eg whether animals could detect  small particles embedded in the agar that alter the agar's surface profile.  Instead, the key experiments here all involve changes to compressibility or shear modulus of agar, while preserving its same surface profile. Indeed the authors use the term "hardness" and "softness" interchangeably with texture, which seems incorrect.    I worry it is at best confusing and at worst outright  incorrect to claim new findings about the
neruobiological basis of detecting food "texture" based on the experiments presented here.

*We thank the reviewer for his insightful comments. Indeed, the experiments performed comprise responses to one aspect of what can be described as texture, and initially we made the decision to use the term ‘texture’ in order to simplify the findings. However, we agree that this may be misleading. In this light, we have made the following changes:*

1) *The title has been revised to “Food hardness preference reveals multisensory contributions of gustatory organs in behaviour and physiology”.*
2) *“texture” has been replaced by “hardness” in the overview of results in the summary section, as well as in the results, figure legends, and discussion to more earnestly reflect the experiments performed.*

Similarly, the analogy to the human tongue probably has to be tempered.

*The comparison to the human tongue has been removed from the discussion section.*

Minor:

- It seems overly strong to claim that "GOs do not appear to contribute to olfactory or light sensing" (line 223) after only testing out one odor (ethyl acetate). This claim should be tempered.

*We have changed the language to emphasise the absence of a phenotype to the specific stimuli tested in order to accurately reflect the results (line 278).*

*The sentence reads as follows: “Thus, we conclude that ablation of GOs are not critically required for driving light avoidance or olfactory attraction to Ethyl acetate.”*

-To help the reader it would be useful to add an x axis label in fig 1B and yaxis labels in figures 2C and 3B.

*We thank the reviewer for this suggestion. We have added “Agarose %” as the X-axis label to figure 1B, and “condition” as the label to figures 2C and 3B.*

-Also it would be helpful to specify that it is water that is on an off in the xaxis of figure 3EandD

*We have added “H2O flow” labels to figures showing responses to water stimulation traces*

Reviewer #3:
Major comments:
1) I do not fully agree with the selection of genetic controls in a few of the individual experiments.
In Fig.2 two genetic controls are used: "GO control" and "rpr control" (I assume that the controls are GO/+ and rpr/+, the information is not provided) alongside with the experimental group "GO>rpr". That is a common standard in the field. However, Fig.3B provides data on different RNAi lines expressed in GO, but only w1118 is used as a control (not quite sufficient in my eyes). Further, in Fig.4D the authors decided to use GO (I assume GO/+, the information is not provided either) as a control in the first 3 panels, while in the last experiment of Fig.4D "GO control" and "rpr control" were used again. In my opinion the selection of genetic controls should not vary between the experiments and only the common standard (GAL4/+ and UAS/+) is sufficient for proper evaluation of the data. In this particular case, I don't think the results of the two data sets will change, but it would be beneficial to completely rule out an effect of the genetic background by the standard
control.

*We thank the reviewer for pointing out these discrepancies, and have made a number of adjustments. We have indeed for all experiments back-crossed the transgenic lines with the respective genetic background (either w1118 or yw). We apoplogize for not clarifying the correct genotypes for the behavioral experiments. In this light, we have now modified the labeling of all controls in the figures and describe them in the respective figure legends.*

2) The presentation of data in Fig.3C,D is not entirely convincing to me, although these results are of high importance. The authors state "we found that 3-4 neurons in the TOG respond to this stimulus with a reduction of fluorescence, indicating a silencing effect of mechanical stimulation". I have a few points here: Why 3-4? 4 neurons are presented in D-E. Please indicate why two curves are presented each (individual measurements?), but only one for CM1 - what is the difference here?

*We are grateful for the question and suggestions posed by the reviewer here. The use of “3-4” stems from the observed variations between recordings, which were only used to spot the phenomenon, rather than to characterise any specific mechanisms. Indeed, the traces shown in figure 4D show two individual traces from each neuron, sorted vertically by response magnitude. This has now been clarified in the manuscript. These traces are used to show the presence of the response rather than to quantify the findings. For CM1 we have also included two representative traces.*

Why is the time frame of the CM1 neuron so different (on-off duration) to the other neurons? Wouldn´t it be worthwhile to quantify the silencing effect (ΔF/F0) to see if there are differences in the effect depending on the type of neurons presented?

*Thank you for pointing out this discrepancy. The timeframe of stimulation is the same for all recordings, and the displayed traces are scaled for the sake of clarity.*

*The difference between the time periods of stimulation is thus simply due to scaling differences during figure preparation (CM1 previously in panel 4E, with a different scale than 4D) . This has now been corrected in figure 3. Cell-identity based differences are further demonstrated in figure 4B.*

L277: Why is only data shown on sucrose stimulation for CM1? It would be useful for the reader to see the data showing that M_ACL, M_CL1 and M-CDL do not respond to sugar. Why are single traces presented in Fig.3 (and Fig.4C), while data for the
other imaging experiments are presented in mean + SEM(SD)?

*We thank the reviewer for pointing this out. We can understand that the presented data seemed incomplete, despite the outlined results. We have now changed figure 3 to include the individual traces of CM1, as well as the sucrose traces for the 3 other identified cells. We have also emphasised that the data presented in figure 3D and 3E were used to uncover a response, rather than to characterise it.*

Minor comments:
1) Line 162-164: "Here, we observe that larvae appear to prefer the specific softness range between 0.5% and 1.5% agarose, with softer and harder substrates elicit aversion. Can the authors really argue that the conc. of agarose below 0.5% and above 1.5%, respectively, are indeed considered as "aversive stimuli"? This is an interesting observation; however, it might only be a preference for 0.5%-1.5% withou t the other concentrations be aversive. Could the authors provide any data on showing that aversion is triggered, like more head casts, higher crawling speed etc. on agarose conc. outside of the preferred range that would indeed point on aversion?

*Thank you for noting this. Indeed, here we erroneously worded the results to suggest that aversion was triggered, when in fact the results only demonstrate a preference for 0.5%-1.5% agarose when compared with excessively soft or excessively hard substrates. We have now changed the wording to reflect this (line 207-208)*

2) Figure 1C' elegantly shows that ablation of GOs reduces the preference for 1% over 2.5% agarose as well as the preference for 1% over very soft 0.1% agarose. However, it appears that larvae do not only lose the preference for 1%, but also show a slight preference for 0.1%, which would even illustrate a shift in valence. Could the authors provide statistical tests against chance level (whether larvae really prefer 0.1%) and comment on how this might fit into their model?

*Thank you for pointing this out. We have now performed an OSS test on the data presented in figure 2C and C’ and indeed we find that the preference for very soft substrate is statistically significant. We have added the full statistical analyses in supplementary table 3 and addressed in the respective portions of the results.*

3) Figure 2C': Why was a different assay used here? The data presented in Fig.1 and Fig.2 C are based on a choice test where different concentrations of agarose are presented on a plate containing two halves of the substrates. However, Fig.2C' uses a different choice test with two circles inside each other. Are they comparable?

*We thank the reviewer for pointing out this mistake. Indeed, all hardness preference experiments were performed as described in the methods (i.e., concentric circles). The results described in lines 201-202 are meant to say “one half of the plate area” rather than “one half”. This has been corrected in the text.*

Furthermore, the data are presented here as a preference for 1% over 2.5% (positive values), whereas they are presented as an avoidance of 2.5% in Fig.1B' (negative values), which is a little confusing.

*Thank you for pointing out that this appears confusing. We have now inverted this graph in figure 2C’ to be consistent with the concept of 2.5% agarose avoidance*

4) L125-127: "We observed that freshly-cut fruits, except banana, are significantly harder than even the hardest (2.5%) agarose concentration in terms of the compression modulus". Figure 1A does not provide any indications for statistical comparisons. Please add.

*We thank the reviewer for pointing this out. In the initial text, we incorrectly use the term “significant” to point out an empirical difference rather than a statistical one. Upon performing statistical analysis, we found no statistically significant difference between the hardness of 2.5% agarose and freshly cut fruits, except banana which was significantly softer. We have now presented this analysis in Figure 1B’’ and adjusted the text to accurately reflect the lack of statistical significance (line 134).*

5) L150-151: Please add more information about the amount/concentration of sugar added to the agarose.

*We have clarified that the doping used was 500mM sucrose, in addition to providing data for other sucrose concentrations tested (10mM and 1M) in figure S4.*

6) L152 / Fig.1B: What does "% ingestion" mean? This is not clear to me.

*We have clarified in the text and in the figure legend that “% ingestion” signifies the percentage of animals from groups of 30 per N for which we observed blue-dyed agarose in the gut. We have now also modified the method section accordingly to provide this information clearer (line 556)*

7) L153-154: "This indicates that there is a specific hardness threshold at which larvae are either unable or unwilling to ingest it". Can the authors rule out that this is primarily true for the experiment done here with the specific concentration of sugar added to the substrate? What will happen if the conc. of sugar added is suboptimal? Does the threshold change?

*This is indeed an intriguing point. We have now performed the same ingestion assays using 10mM and 1M sucrose concentrations and do not find that this pattern changes, and we have presented these data in figure S4, as well as addressing this in the text (lines 188-190)*

8) Some references may be worth adding, e.g.  Apostolopolou et al. 2014 (doi:10.3389/fnbeh.2014.00011), as they show (in a slightly different context) that agarose concentration affects larval behavioural performance.

*We have added this reference (line 55) as well as ones additionally pointed out by other reviewers, to the text. Thank you for this suggestion*.


Reviewer #4: The authors of the paper "Food texture preference reveals multisensory contributions of gustatory organs in behaviour and physiology" investigate the role of neurons in the gustatory organ in integrating mechanosensory and taste information. First, they convincingly demonstrate a preference of larval Drosophila to navigate towards substrates with specific hardness, which matches their ability to ingest food with this hardness.  They then show through cell ablation, RNAi and eventually calcium imaging how specific neuronal cell types respond to either mechanical cues or multi-modally integrate multiple cues. Overall, the paper represents an interesting investigation that is relevant for understanding how larvae select appropriate food sources. This paper adds to the increasing evidence that multi-modal neurons are common (at least in invertebrate models) and likely play an important role in foraging decisions.  

While the paper is overall convincing, I have some major and minor concerns detailed below.

Major concerns:
- Overall, methods could b e described in a touch more detail which would help the readers. I detail a few examples below where the lack of detail on what was quantified is confusing to the reader.

- decay is relative to ripeness etc., which the authors already acknowledge in the discussion. I suggest normalizing the compression modulus in panel A''' and/or adding a normalized panel as well as a sentence describing the limitation of estimating a 'start date' for the decay in the results section. In a similar vein, this sentence should also be rephrased: "This correlates to the hardness of pear and pineapple after 3-4 days of decomposition, highlighting that fruit hardness may provide sensory cues about the state of decomposition."

*We thank the reviewer for noting this. We have now added a panel to show the relative hardness of each fruit normalised to the day 1 values (figure 1A’’ right) and relative agarose hardness normalised to 2.5% hardness values (figure 1B’). In addition, we have added text to emphasise that many factors may cause differences in hardness in this experimental paradigm, thus the results should not be treated as representative of all fruit hardness (lines 136-139). We have also reworded the suggested line to tone down the speculation, it now reads: “suggesting that hardness may present as an additional sensory cue” (line 210-211)*

Ingestion assay: The ingestion assay should clarify if the % ingestion denotes the fraction of animals that showed blue dye (assessed visually?) or if this is a quantitative measure of the amount of ingested dye? This is unclear from the y-label and the results section.

*We thank the reviewer for highlighting this lack of clarity. Indeed, the % of animals ingested is assessed visually, as shown in figure 1. We found no ambiguity in assessing if an animal was or was not containing blue dye. We have clarified this in the results section, and we have also emphasised this point, and that the counting was done in a double-blind manner, in the methods section.*

Larval taste organ identification:
Fig. 2A - what are the gray points? Is this data from all head neurons or only DOG/VO?

*We thank the reviewer for highlighting this lack of clarity. We have pointed out in the text (line 234) and figure legend that the cells presented in this figure are in fact filtered by pebbled expression, indicating that these are all sensory neuron cells*

My major concern about the paper is about the calcium imaging.
Live calcium imaging in Fig. 3:
I am very confused if the traces shown represent a mean or a sample and error bars are not shown? Without any error bars or quantification, we can not assess if the changes in intensity are meaningful, robust and significant!

*We thank the reviewer for pointing out this lack of clarity. The traces shown represent the responses of the neurons across 2 individual recordings, and are only used as preliminary indications of the mechanosensitive phenomenon. We have now clarified this in the figure legend and text (line 335-337). Due to the low number of individual recordings, we opted to not include statistical analysis here as it would not provide useful information. Instead, we chose to investigate this phenomenon in individual known neurons.*

The methods section for the calcium imaging does not describe how the data were analyzed, in neither the single cell or multi-cell imaging case.

*Thank you for pointing out this omission, indeed this was in error. We have now added an analysis section (lines 581-590) in the materials and methods, pointing out that the analysis was perfomed as previously published by our laboratory in Maier et al., 2021 eLife. A summary of the analysis has also now been provided.*

In addition, the authors should either make use of the ratiometric indicator to remove artifacts from changing the flow rate (which may move or compress the cells). Alternatively, please include a supplementary figure showing how the fluorescence in the red channel changes. This is particularly important for the mechanical stimulus, but should be presented for all calcium data, especially given the large spread in dF/F0 values in Figure 4.

*Thank you for making this suggestion. While we assumed that the signal would not change, we did not initially analyse the signal in the red channel. We have now added a supplemental figure (supplemental figure 3) which includes these analyses: (A) RFP channel traces from pain-gal4 neurons analysed in figure 3D and E, showing stable fluorescence values across time; (B) RFP channel fluorescence from C6 recordings analysed in figure 4, similarly showing no fluorescence fluctuation in both mechanical and chemosensory recordings.*

The term 'Live calcium imaging' for the data in Fig. 3 appears a bit misleading, as the preparation uses dissected heads. I suggest rephrasing this or at least acknowledging the difference between an intact animal and a dissection in the appropriate results section.

*We thank the reviewer for pointing this out. We have rephrased the term “live calcium imaging” with “in-situ calcium imaging”. Indeed, we used the terminology previously used in similar experiments (van Giesen et al., 2016b; Maier et al., 2022), however we agree with the reviewer that “live” may be misleading in the wider context of similar experiments performed in Drosophila and beyond.*

- In Fig. 4D, it seems the response of the RNAi of Gr63/Gr21 in C6 does have an effect in mechanosensation, namely the neuron seems to be inhibited much longer compared to wt (panel 4B). What is the interpretation of this difference (if it is significant)?

*We thank the reviewer for pointing this out. While we mainly focused on the initial reduction in fluorescence upon mechanical stimulation, it is indeed notable that the refraction of the neuron to the depolarised and/or resting state is affected upon the loss of CO2 receptors. We have now addressed these results in the discussion section (lines 431-434) to point out this difference.*

minor concerns:
Statistics:

The data is overall very convincing and the below are points mainly to help the reader assess the work.
In many figures it is hard to quickly see the sample sizes due to the color of the individual points. It would be helpful to have the N number denoted in the caption or figure itself, e.g., in Fig. 1B-B''', as well as for the calcium imaging in Fig. 4.

*Thank you for pointing out this lack of clarity. We adjusted the figures so that individual points are now in black and thus have a higher contrast and clarity. We have also now added N values in all relevant figures to clarify what was done, in addition to the information provided in the captions.*

For Figure 1B, please describe what was tested e.g., based on the test it seems that group difference was assessed, rather than  significant ingestion relative to 0. It would be useful to the reader to be explicit about this.

*We thank the reviewer for this suggestion, and have now added this information in the figure legend, outlining that the the ingestion was assessed by inspecting 30 larvae (per N) for presence of blue dyed agarose in the gut.*

In Figure 2C', the authors state that larvae show a preference for softer substrates, however the chosen test (One-way ANOVA) tests for group-mean differences as indicated in the figure. To support this statement, the authors should test if the ablated data shows a significant preference compared to a preference index of 0. ('Larvae lose preference for the softer 1% agarose versus 209 harder 2.5% agarose, in addition they start to prefer excessively soft 0.1% agarose')

*We thank the reviewer for this suggestion, and indeed a similar point was raised by another reviewer. In this light we have added a table (supplemental table 3) showing results of One sample t and Wilcoxon statistical analyses of data presented in figure 2C and C’ vs a hypothetical mean of 0.*
